# Expression of a Chimeric Gene Encoding Insecticidal Crystal Protein Cry1Aabc of *Bacillus thuringiensis* in Chickpea (*Cicer arietinum* L.) Confers Resistance to Gram Pod Borer (*Helicoverpa armigera* Hubner.)

**DOI:** 10.3389/fpls.2017.01423

**Published:** 2017-08-21

**Authors:** Alok Das, Subhojit Datta, Shallu Thakur, Alok Shukla, Jamal Ansari, G. K. Sujayanand, Sushil K. Chaturvedi, P. A. Kumar, N. P. Singh

**Affiliations:** ^1^Division of Plant Biotechnology, ICAR-Indian Institute of Pulses Research Kanpur, India; ^2^Division of Crop Protection, ICAR-Indian Institute of Pulses Research Kanpur, India; ^3^Division of Crop Improvement, ICAR-Indian Institute of Pulses Research Kanpur, India; ^4^ICAR-National Research Centre on Plant Biotechnology New Delhi, India

**Keywords:** transgenic chickpea, *Cicer arietinum* L., genetic transformation, *Agrobacterium*, *Helicoverpa armigera*, *cry1Aabc*, *Bacillus thuringiensis*

## Abstract

Domain swapping and generation of chimeric insecticidal crystal protein is an emerging area of insect pest management. The lepidopteran insect pest, gram pod borer (*Helicoverpa armigera* H.) wreaks havoc to chickpea crop affecting production. Lepidopteran insects were reported to be controlled by *Bt* (*cryI*) genes. We designed a plant codon optimized chimeric *Bt* gene (*cry1Aabc*) using three domains from three different *cry1A* genes (domains I, II, and III from *cry1Aa*, *cry1Ab*, and *cry1Ac*, respectively) and expressed it under the control of a constitutive promoter in chickpea (*cv*. DCP92-3) to assess its effect on gram pod borer. A total of six transgenic chickpea shoots were established by grafting into mature fertile plants. The *in vitro* regenerated (organogenetic) shoots were selected based on antibiotic kanamycin monosulfate (100 mg/L) with transformation efficiency of 0.076%. Three transgenic events were extensively studied based on gene expression pattern and insect mortality across generations. Protein expression in pod walls, immature seeds and leaves (pre- and post-flowering) were estimated and expression in pre-flowering stage was found higher than that of post-flowering. Analysis for the stable integration, expression and insect mortality (detached leaf and whole plant bioassay) led to identification of efficacious transgenic chickpea lines. The chimeric *cry1Aabc* expressed in chickpea is effective against gram pod borer and generated events can be utilized in transgenic breeding program.

## Introduction

Chickpea (*Cicer arietinum* L.) is an important grain legume and holds third position in food legume production worldwide. It is an annual, self-pollinated, diploid (2n = 2x = 16) pulse species of *Fabaceae* family, with genome size of approximately 738 Mb (Varshney et al., 2013). India alone accounts for 70–75% of total global chickpea production and currently is the largest consumer and importer of chickpea. In India, chickpea is grown in an area of 8.5 m ha with average annual production of 8.83 m t (FAOSTAT, 2014). Two main types of chickpeas grown are small-seeded *desi* primarily consumed in the Middle East and Southeast Asia and larger-seeded *kabuli* is a valuable global commodity. Chickpea seeds are rich in dietary protein (20–25%), and crop helps in soil fertility management through symbiotic nitrogen fixation. Despite its nutritional value and important role in value addition, its productivity are stagnating (<1000 kg/ha) owing to several biotic and abiotic stresses like insect pests, *Fusarium* wilt, *Ascochyta* blight, heat, drought, salinity, and unpredictable climatic conditions. Insects particularly, gram pod borer (*Helicoverpa armigera* Hubner) are major pest which attack chickpea starting from first fortnight after sowing, voraciously during flower and pod development stages resulting in yield loss of up to 20–30% annually.

Farmers turn to prophylactic insecticidal sprays for control of these insect pests which are expensive and often have adverse effect on environment and human health. Hence, management of pod borers remains a major concern for farmers and the situation is predicted to aggravate by global warming and climate change that would influence activity, diversity, distribution, and population dynamics of the insect pest (Sharma, 2010). Conventional breeding approaches for insect resistance have limited success due to lack of resistance source and incompatibility with the wild relatives (Nene et al., 1990). Thus, development of pod borer resistant chickpea varieties by genetic engineering, utilizing *Bt* gene is an important endeavor. There are several successful reports of genetic transformation of chickpea for various traits (Das and Parida, 2014). Successful transformation of chickpea for insect resistance were reported utilizing *Bt* genes for pod borer resistance (Kar et al., 1997; Sanyal et al., 2005; Acharjee et al., 2010; Mehrotra et al., 2011; Ganguly et al., 2014; Chakraborty et al., 2016), bean alpha-amylase inhibitor 1 (*α-ai1*) for bruchid (*Callosobruchus* spp.) resistance (Sarmah et al., 2004; Ignacimuthu and Prakash, 2006) and lectin (ASAL) against aphids (Chakraborty et al., 2009). However, Bt-transgenic plants often show variable resistance responses against insect pest, due to various factors, *inter alia*, lower expression and late season decline in expression of *Bt* toxin (Adamczyk and Summerford, 2001; Kranthi et al., 2005; Olsen et al., 2005a,b). Transgenic plants with reduced expression or efficacy would substantially increase the possibility of developing resistant insect population (Fitt et al., 1998). Hence, the challenge is to develop insect resistant lines by increasing and stabilizing the level of gene expression in target tissues or use multiple/novel/fusion protein to confer complete protection against gram pod borer.

The insecticidal crystal proteins (ICPs) of Cry1 class impart resistance against insects of Lepidopteran family. Cry1 proteins are reported to have three domains: a hydrophobic and amphipathic seven alpha-helix bundle forming domain I, implicated in pore formation; a triple anti-parallel beta sheets forming domain II for specificity determination and receptor binding and beta sheet sandwich forming domain III involved in receptor binding and protease protection (Kumar et al., 1996). Synergistic activity among different ICPs to augment insect pest resistance spectrum was reported earlier (Xue et al., 2014). Due to high specificity for unique receptors on the membrane of the insect gut epithelial cells, ICPs are reported harmless to non-target insects and are compatible with integrated pest management programs. Generation of recombinant fusion proteins and domain swapping are emerging areas to improve protection spectrum against insect pest (Saraswathy and Kumar, 2004). In the present study, we report development and characterization of transgenic chickpea lines with the domain shuffled chimeric gene, *cry1Aabc* for protection against gram pod borer (*H. armigera* H.).

## Materials and Methods

### Experimental Material and Plant Tissue Culture Conditions

The breeder’s seed of *desi* chickpea variety, DCP92-3 was used for genetic transformation experiments. The protocol utilized modified MS medium consisted of MS salts (Murashige and Skoog, 1962), B_5_ vitamins (Gamborg et al., 1968), 3% sucrose and 0.8% Agar (pH 5.8). The growth regulators, cytokinins [6-benzyl amino purine (BAP), 6-furfurylaminopurine (kinetin), thidiazuron (TDZ) and 2-isopentenyl adenine (2iP)], gibberellic acid (GA_3_), three organic additives [L-cysteine, L-glutamine, and L-arginine (Duchefa Biochemie, Haarlem, the Netherlands)] and antibiotics kanamycin monosulfate, cefotaxim and rifampicin and acetosyringone (Sigma Aldrich, United States) were added to different medium after filter sterilization or autoclaving. The cultures were incubated at 23 ± 2°C under a light regime of 16 h light (100 μmol/m^2^/s) provided by cool white fluorescent lamps and 8 h dark.

### Binary Vector and Genetic Transformation

For genetic transformation, the binary vector pBinAR originally derived from pBin19 (Hofgen and Willmitzer, 1990), harboring the chimeric *Bt* δ-endotoxin gene, *cry1Aabc* and *neomycin phosphotransferase II* (*nptII*) as the plant selectable marker gene (within T-DNA) was used (**Supplementary Figure S1**). The *cry1Aabc* gene was constructed by fusing three different domains (I, II, and III) from three different *Bt* genes: *cry1Aa*, *cry1Ab*, and *cry1Ac*, respectively. Notably, the synthetic gene was designed based on codon usage of plants and deletion of mRNA destabilizing features prevalent in the native gene sequences (Murray et al., 1989; Hebsgaard et al., 1996; Liu et al., 2005; Chang et al., 2013; Dassi et al., 2014). The re-constructed binary vector (14,350 bp), after confirmation by sequencing, was mobilized into *Agrobacterium tumefaciens* strain, EHA105 (Hood et al., 1986) and used for genetic transformation studies. *Agrobacterium* mediated genetic transformation was attempted in *desi* chickpea cultivar, DCP92-3. *Agrobacterium tumefaciens* strain (EHA105) was grown overnight at 28°C in Yeast Extract Mannitol (YEM) broth containing 50 mg/L Kanamycin monosulfate and 10 mg/L Rifampicin. The bacterial cells (0.6–0.8 O.D. at 600 nm) were harvested by centrifugation and resuspended in half strength of MS medium containing 100 μM Acetosyringone.

Asceptically, overnight soaked seeds of chickpea were de coated and inoculated in modified MS medium (MS salts + B_5_ vitamins + 2.0 mg/L 2iP + 0.43 mg/L Kinetin + 0.88 mg/L TDZ + 3% sucrose + 0.8% agar, pH 5.8) for germination and induction of axillary meristematic region for 7 days. Axillary meristem explants (AMEs) were prepared from healthy germinated seedlings by removing axillary buds up to the base and cuts were made to remove the plumule and radical tips up to the hypocotyl and epicotyl regions. The prepared explants were infected with prepared *Agrobacterium* culture for 10 min, and the infected explants were inoculated in co-cultivation medium (MS + B_5_ vitamins + 1.0 mg/L BAP + 200 mg/L L-cysteine + 0.60 mg/L L-glutamine + 0.70 mg/1 L-arginine + 3% sucrose + 0.8% agar, pH 5.8) for 72 h in diffused light. Initially, the cotyledons were removed from explants with multiple shoots and transferred to shoot induction medium (MS salts + B_5_ vitamins + 0.43 mg/L Kinetin + 1.0 mg/1 2iP + 250 mg/L cefotaxime + 100 mg/1 Kanamycin + 3% sucrose + 0.8% agar, pH 5.8) medium and incubated for 10–14 days. Subsequently, at every sub culturing, individual shoots were separated from clumps and transferred to elongation medium (MS salts + B_5_ vitamins + 1.0 mg/1 GA_3_ + 100 mg/L kanamycin + 4% sucrose + 0.8% agar, pH 5.8) medium for elongation and antibiotic selection. Shoots were subcultured every 10–14 days in the same medium for 4–5 cycles to obtain green healthy shoots, after removal of chlorotic/dead shoots. The elongated and healthy shoots were used as scions and grafted on to non-transgenic root stocks (DCP92-3) initially grown on soil matrix. The grafts were covered by polythene bags and transferred to Containment Facility for acclimatization and hardening. The polythene bags were punched every alternate days to acclimatize to natural conditions. After 7–10 days of grafting, the polythene bags were carefully removed and the grafted plants were exposed to Contained Conditions for normal growth and development. Seeds were harvested from mature fertile plants (**Supplementary Figure S2**).

### Confirmation of cry1Aabc Gene in Putative Transgenic Plants

Total genomic DNA was isolated from young leaves of putative transgenic chickpea plants using customized plant DNA isolation Kit (DNeasy Plant Kit, Qiagen, Hilden, Germany). The presence of *cry1Aabc* gene in putative transgenic chickpea plants were confirmed by PCR using the gene specific primers [A4F- 5′-CCTTGTACAGAAGACCCTTCAATATC-3′ (forward) and A4R-5′-TCTATTCTGAATGTTATTTCCACTGC-3′ (reverse)]. The thermal amplification was carried out using 1X Taq buffer, 200 μM dNTP mix, 10 pM each primer, 150–200 ng DNA template and 1 U *Taq* DNA polymerase (Merck GeNei, Bangalore) in 20 μl volume. PCR reaction was performed in thermal cycler (BioRad, United States) with the following program: initial denaturation at 94°C for 5 min, followed by 35 cycles of denaturation at 94°C for 60 s, annealing at 60°C for 60 s and extension at 72°C for 45 s, with a final extension at 72°C for 8 min. Products were resolved on 1.0% agarose gel stained with Ethidium Bromide (EtBr) in 1X TAE buffer. Images of gel were documented in Gel Documentation system (BioRad Gel Doc XR, United States).

For genomic Southern hybridization, DNA was isolated from leaves of putative transgenic and *wild-type* (WT) chickpea plants (Dellaporta et al., 1983). Isolated DNA was digested with restriction endonuclease, *Hind*III (which restricts the T-DNA at one site near the termination of *cry1Aabc* gene) and double digested with *EcoR*I and *Hind*III (which release the entire *cry1Aabc* cassette) separately. The digested products of each line were electrophoresed in a 1.0% agarose gel and then blotted onto Hybond N+ membrane. The digoxygenin (DIG) labeled probe, specific to *cry1Aabc* gene was synthesized using DIG DNA labeling kit. Labeling, hybridization and detection were performed using DIG labeling Kit with the chemiluminiscent substrate, disodium 2-chloro-5-(4-methoxyspiro {1,2-dioxetane-3,2’-(5′-chloro)tricyclo[3.3.1.1]decan}-4-yl)-1-phenyl phosphate (CDP-*Star*), according to manufacturer’s instructions (Roche Diagnostics GmbH, Mannheim, Germany). Hybridization was carried out at 65°C overnight in hybridization oven (Stuart, United Kingdom). Stringent washes were performed using a primary wash buffer at 65°C for 20 min and a secondary wash buffer at 25°C for 10 min. The signal was detected on an X-ray film (Roche Diagnostics GmbH, Mannheim, Germany) after an exposure of 10 min. The X-ray film was scanned using Pharos FX Plus Molecular Imager (BioRad, United States).

### Estimation of Cry1Aabc Protein in Transgenic Chickpea Plants

For detection and estimation of Cry1Aabc protein in putative transgenic chickpea progenies, total soluble protein (TSP) was isolated from leaf tissues using bicarbonate buffer (15 mM Sodium carbonate, 35 mM Sodium bicarbonate, 0.1% Triton X 100, 0.05% Tween 20, 0.5% Sodium azide, 1% polyvinylpyrollidone (PVP) and 1 mM phenylmethanesulfonylfluoride (PMSF) (Sigma Aldrich, United States), added to ELISA plates (Greiner Bio One, Germany) and incubated overnight at 4°C along with controls and blank. The wells of ELISA plate were blocked using 1% bovine serum albumin (BSA) (Sigma Aldrich, United States) for 90 min and washed with phosphate buffer saline Tween (PBST) at 37°C, thrice. Cry1Ac specific primary antibody were added to each well and incubated for 90 min at 37°C. Alkaline phosphatase conjugated secondary antibody were added to wells for binding with primary antibodies, followed by washing thrice with PBST and subsequently incubated for 90 min at 37°C. After incubation, free secondary antibodies were washed with PBST and PBS. Alkaline phosphatase specific substrate, *para*-nitrophenylphosphate (pNPP) (Sigma Aldrich, United States) was added to the plate and incubated for 30 min at 37°C. The absorbances of the samples were measured at 405 nm wavelength in ELISA reader (Spectra Max, Molecular Devices, United States).

Protein was quantified at different developmental stages from different target tissues (leaves, immature seeds, and pod walls) in selected chickpea events. Total protein was isolated from leaves at 153 days after sowing (DAS) at T_3_ stage and at T_4_ stage, isolation was done both at pre-flowering (88 DAS) and post-flowering (116 DAS) stages. For tissue specific expression studies, total protein was extracted from young leaves, pod walls, and immature seeds (129 DAS). Quantity of Cry1Aabc protein was estimated in chickpea progenies in terms of ng/mg of TSP using quantitative ELISA Kit (Envirologix, United States).

For western hybridization, total protein was isolated from young chickpea leaves (T_3_ stage: 164 DAS; T_4_ stage: 145 DAS), using bicarbonate buffer [15 mM Sodium carbonate, 35 mM Sodium bi carbonate, 0.1% Triton X 100, 0.05% Tween 20, 0.5% Sodium azide, 1% PVP, 1 mM PMSF, 1% plant preotease inhibitor cocktail (PPIC)]. Quantification of TSP was performed using Bradford reagent (Sigma Aldrich, United States) (Bradford, 1976) and 30 μg protein was fractionated on 15% acrylamide gel with 10% SDS and blotted on to nitrocellulose membrane (BioRad, United States) by wet transfer. Cry1Ac specific primary antibody (Envirologix, United States) was used and horse radish peroxidase conjugated secondary antibody (Sigma Aldrich, United States) were used for detection. The blot was developed in X-ray films using chemiluminiscent substrate, luminol (Roche Diagnostics GmbH, Mannheim, Germany) in developer and fixer (Kodak, Germany). The X-ray film was scanned using Pharos FX Plus Molecular Imager (BioRad, United States).

### Insect Bioassay

The young and tender chickpea leaflets were clipped from three transgenic lines and control (DCP92-3) (40–60 days old) and were immediately washed with sterile water to remove any inert material adhering to it. Initial weights of cleaned leaflets were taken and placed in 50 ml vial containing 2% water agar slants with 0.1% (w/v) sorbic acid to avoid any mold growth in media. The freshly hatched 24 h old neonates reared on artificial diet (Olsen and Daly, 2000) from National Transgenic Testing Facility were used for insect bioassay against transgenic chickpea lines. To each container three neonates were released carefully on the chickpea leaflet with the help of camel hair brush. The lids were perforated with a paper pin to ensure air circulation. Similarly, two other replications were also prepared. These vials were arranged in a tray and were kept inside environmental chamber (Model: CHM10 Plus, REMI) at 25 ± 5°C and 70 ± 5% RH. The experiment was conducted in completely randomized design (CRD). The percent mortality was calculated from 24 to 168 h on daily basis (Abedi et al., 2014). Average mortality was calculated for the corresponding event. After 7 days, the remaining leaflet weight was recorded to find out the amount of leaf consumed (g) by the neonates. Similarly, the larval weight gain (g) on the 7th day was recorded to calculate the larval weight gain. The percent mortality data and weight of leaf consumed by larvae were subjected to statistical analysis in SAS 9.2 (SAS Institute, Inc., Cary, NC, United States) by using ANOVA procedure with *post hoc* test Duncan’s multiple range test (DMRT). *P* < 0.01 was considered to be statistically significant.

For whole plant bioassay, transgenic chickpea plants (IPCa2) were covered with a cylindrical plastic cage having opening at top and bottom. A small window covered with muslin cloth was provided at a height of 20 cm to ensure air movement inside the cage. Eight 24 h old neonates were released to each transgenic chickpea plant and the top opening was sealed with a fine muslin cloth. The percent mortality was recorded up to 7 days after release and data was analyzed by *t*-test for two samples assuming equal variance (SAS Institute, Inc., Cary, NC, United States).

## Results

### Construction of Chimeric Bt Gene and Production of Transgenic Plants

A plant codon-optimized chimeric *Bt* ICP gene *cry1Aabc* was constructed with nucleotide sequences that encode three different domains I, II, and III from three different ICPs *viz.*, *cry1Aa, cry1Ab, and cry1Ac*, respectively. The chimeric protein differs from Cry1Ac protein (truncated) with respect to five amino acids in the first two domains (I and II) (Adang et al., 1985) (**Table 1**). The codons were optimized for plant expression, A+T content in nucleotide base composition and elimination of sequences like destabilization, inappropriate polyadenylation, degradation and presence of RNA splice sites, etc. (**Supplementary Table S1**).

**Table 1:** Features of *Cry1Aabc* protein *vis a vis cry1Ac* protein.

Features	*Cry1Aabc*	*Cry1Ac*^∗^
Domain I	148 (Leu)	148 (Phe)
Domain II	248 (Ser)	248 (Pro)
	280 (Thr)	280 (Ser)
	290 (Glu)	290 (Arg)
	313 (Glu)	313 (Tyr)
Domain III	–	–


*^∗^Truncated *Cry1Ac* (Adang et al., 1985).*

*Agrobacterium tumefaciens* mediated genetic transformation was attempted in 7,892 chickpea AMEs and six putative transgenic (kanamycin resistant shoots) lines were established in the Transgenic Containment Facility with the transformation frequency of 0.076%. All the established putative transformants (T_0_) were screened through PCR using gene specific primers, and an amplification product of 440 bp were found. Selfed seeds from established plants were harvested and re-sown in the Containment Facility for generation advancement and molecular analyses. These lines were advanced to T_2_–T_4_ stages and detailed molecular characterizations of three events *viz*. IPCa1, IPCa2, and IPCa3 were taken up subsequently.

### Molecular Characterization of Transgenic Lines

**FIGURE 1:**
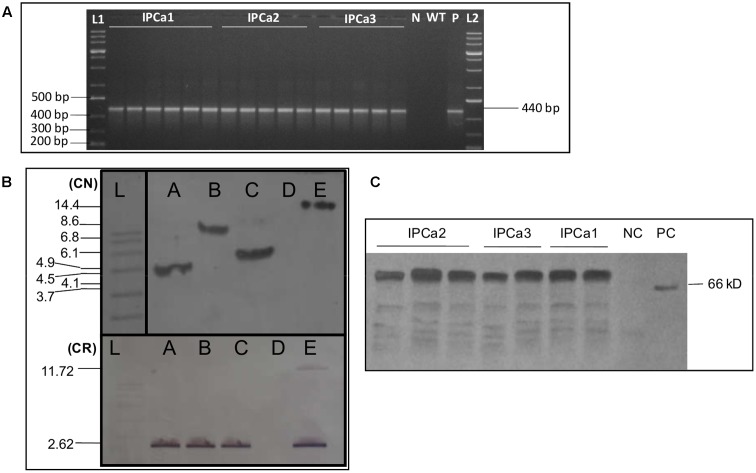
Molecular analysis of three transgenic chickpea lines. **(A)** PCR analysis of transgenic chickpea progenies. Lanes are as follow, L1: 1 kb DNA Ladder (Thermo Scientific, United States); IPCa1 (Lanes 1–6): progenies from IPCa1; IPCa2 (Lanes 7–11): progenies from IPCa2; IPCa3 (Lanes 12–16): progenies from IPCa3; N: no template control; WT: wild-type (non-transgenic line); P: positive control (Plasmid); L2: 1 kb DNA ladder (Thermo Scientific, United States). **(B)** Southern blot analysis of three events using *cry1Aabc* specific probe in T_4_ generation. Copy number (CN) identification using *Hind*III. Cassette release (CR) (*Cry1Aabc* gene) in transgenic lines using *Hind*III and *Eco*RI. Lanes are as follow, L: DIG labeled mol. wt. marker (Roche Diagnostics GmbH, Mannheim, Germany); A: IPCa1 event; B: IPCa2 event; C: IPCa3 event; D: negative control (non-transgenic DCP92-3); E: positive control. **(C)** Western blot analysis (T_4_ Generation) [Lanes 1–3: IPCa2 progenies; Lanes 4–5: IPCa3 progenies; and Lanes 6–7: IPCa1 progenies; NC: negative control; PC: positive control (Purified Cry)].

PCR analyses of the progenies done during T_2_–T_4_ stages indicated the presence of amplification product of 440 bp in the progenies, derived from three events (**Figure 1A**). To detect the stable integration of *cry1Aabc* gene in three events (T_3_ and T_4_ generation), DNA was pooled from progenies of all the three individual events separately and subjected to Southern blot hybridization. The presence and single locus integration was detected in all the three events. Genomic DNA digested with *Hind*III exhibited single bands in each events corresponding to a unique location in the genome (*ca*. 4.5 kb for IPCa1, 8.6 kb for IPCa2, and 6.1 kb for IPCa3) using gene specific DIG labeled probe. Digestion with two restriction endonucleases (*EcoR*I and *Hind*III) exhibited 2.62 kb of recombinant gene (*cry1Aabc*) released form the genome (**Figure 1B**), indicated intactness of inserted cassette.

Direct antigen coating (DAC) ELISA (qualitative ELISA) was performed to confirm the expression of Bt protein in all chickpea progenies derived from three events at T_3_ and T_4_ generation. Notably, all the analyzed progenies in both the generations exhibited expression. In T_3_ generation, 33 progenies have been found positive derived from all the three events and in T_4_ generation 102 progenies derived from all three events were found positive.

Double Antibody Sandwich ELISA (quantitative ELISA) were performed in all chickpea progenies for the quantification of Bt protein. In T_3_ generation, a total 33 chickpea progenies were analyzed for δ-endotoxin expression is leaf tissues, 153DAS. Overall, protein expression range was 8.57–39.44 ng/mg of TSP. A total 102 T_4_ chickpea progenies (derived from three events) were analyzed for assessing the expression at pre-flowering (88 DAS) and post-flowering stage (116 DAS) using quantitative ELISA protocol. For all the events, the expression at pre-flowering (range: 7.96–12.86 ng/mg TSP) was higher as compared to post-flowering stage (range: 6.11–9.03 ng/mg TSP) Tissue specific expression in pod walls and immature seeds (129 DAS), indicated higher expression in pod walls (av. 10.60 ng/mg TSP), as compared to immature seeds (av. 5.51 ng/mg TSP) (**Table 2**).

**Table 2:** Quantification of *Cry1Aabc* protein at pre flowering and post-flowering stage (T_4_ generation) of different chickpea events.

Event	Pre-flowering	Post- flowering		
		
	Leaf (88 DAS)	Leaf (116 DAS)	Immature seeds (129 DAS)	Pod wall (129 DAS)
IPCa1	10.84 ± 9.22	6.11 ± 2.44	7.11 ± 3.91	10.01 ± 4.22
IPCa2	7.96 ± 3.31	6.51 ± 2.59	4.77 ± 0.97	11.86 ± 3.08
IPCa3	12.86 ± 6.77	9.03 ± 4.76	4.67 ± 0.43	9.94 ± 3.99


The Cry1Aabc protein was detected in all progenies derived from three events using Western blot hybridization. In all the events, ∼66 KDa band was observed in leaf samples harvested post-flowering, without any degradation corresponding to expressed Cry1Aabc protein (**Figure 1C**).

### Insect Bioassay

**FIGURE 2:**
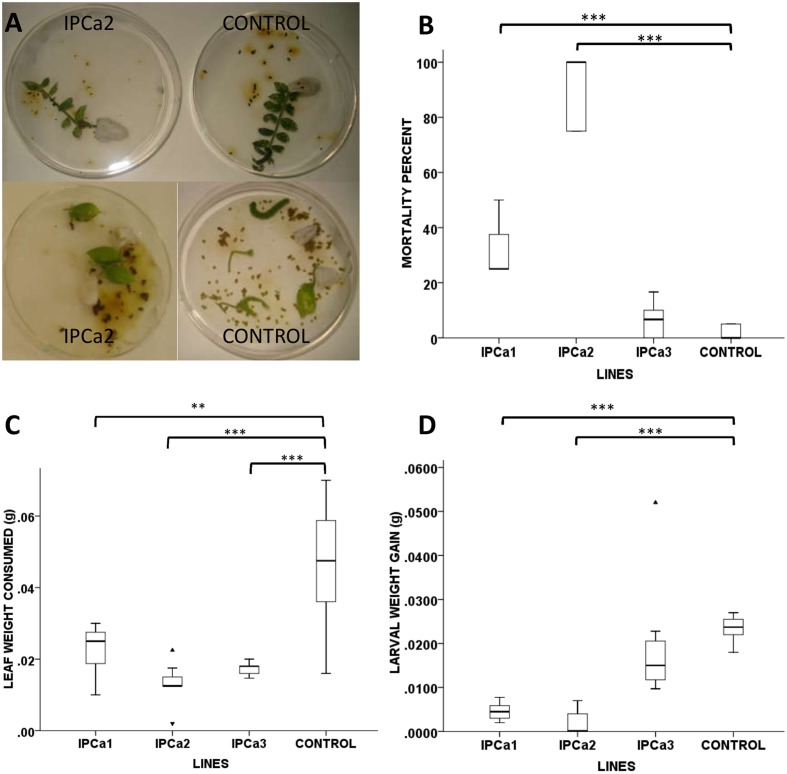
Insect bioassay of transgenic chickpea lines. **(A)** Detached leaf assay (no choice condition) of transgenic chickpea line (IPCa2) and non-transgenic DCP 92-3 (CONTROL). **(B)** Box plot depicting significant higher percent mortality of neonates in transgenic chickpea line (IPCa1 and 2) compared to non-transgenic DCP92-3 (CONTROL). **(C)** Box plot depicting significantly lower average leaf consumed by neonates in all transgenic chickpea lines (IPCa1, 2, and 3) compared to non-transgenic DCP92-3 (CONTROL) (*F* = 12.037, *P* < 0.01). **(D)** Box plot depicting significant lower average weight gain of neonates in transgenic chickpea line (IPCa1 and 2) compared to non-transgenic DCP92-3 (CONTROL) (*F* = 13.482, *P* < 0.001). ^∗∗^*P* < 0.01 and ^∗∗∗^*P* < 0.001. 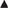 indicate higher outlier and 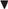 lower outlier.

Three transgenic chickpea lines IPCa1, IPCa2, and IPCa3 were subjected to insect (*H. armigera*) bioassay using 24 h old neonates, in young trifoliate leaves and whole plant bioassay was done using transgenic chickpea line, IPCa2. In detached leaf bioassay (under no choice condition), there was significantly greater larval mortality and lesser defoliation in transgenic lines (IPCa1 and IPCa2) as compared to the control lines (*F* = 118.68, *P* < 0.001) (**Figures 2A,B**). Larval mortality between the controls (DCP92-3) did not differ significantly in the experiments.

**FIGURE 3:**
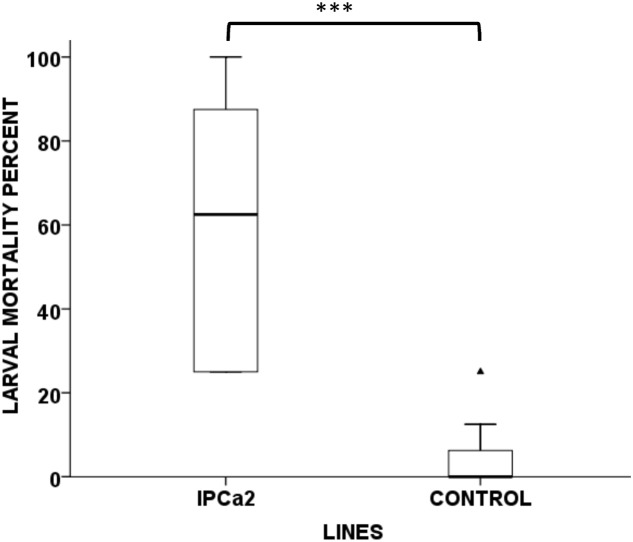
Whole plant bioassay of transgenic chickpea line IPCa2 compared to control. Box plot indicating significant differences between the transgenic line IPCa2 and non-transgenic DCP92-3 (CONTROL) (*P* < 0.001, *F* = 36.954, CD = 19.31). ^∗∗∗^*P* < 0.001 and 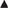 indicate higher outlier.

Average leaf consumed by neonates in transgenic chickpea lines (0.01–0.02 g) were significantly lower (*F* = 12.037, *P* < 0.01) than non-transformed lines (0.04 g), indicating affectivity of the transgenic lines (**Figure 2C**). Further, retarded pattern of growth and development were exhibited in the larvae fed on transgenic lines as compared to control. Average weight gain of larvae fed on transgenic lines IPCa1 and IPCa2 were significantly lower (*F* = 13.482, *P* < 0.001) than the control (**Figure 2D**). In case of whole plant bioassay, *t*-test reveals mortality in transgenic line (IPCa2) was significantly higher (*P* < 0.001) compared to control (**Figure 3**) (**Supplementary Table S2**). In few lines, co-relation could be established between Bt-protein and larval mortality based on detached leaf bioassay, indicating the effectiveness of Cry1Aabc protein in larval mortality (**Table 3**).

**Table 3:** Correlation between efficacy and the amount of Insecticidal *Cry* protein in leaf tissues.

Line	Expression	% Larval mortality
IPCa 1 (A.3.1.11)	10.84 ± 9.88	76
IPCa 1 (A.3.2.12.1)	11.04 ± 5.32	77
IPCa 2 (B.15.2.1.7)	12.81 ± 1.23	75
IPCa 2 (B.15.2.11)	19.14 ± 0.27	100
IPCa 2 (B.15.1.12)	16.34 ± 5.09	89
Control (DCP92-3)	0	0


## Discussion

Chickpeas are protein and mineral rich grain legume that foster sustainable agriculture with smaller carbon footprint. Climate change effects on insect pest spectrum and incidence is the major concern in chickpea, seeds of which serve as protein source for millions. The gram pod borer (*H. armigera* H.), is the most devastating insect pest of chickpea in India. Insect infestation begins a fortnight after germination and become voracious during reproductive stage. Larvae skeletonise leaves leaving only veins and subsequently move to flower buds and green pods. Notably, larvae make circular holes and feed on the developing grains rendering empty pods. The life cycle is completed in 30–45 days with eight generations in a year. With increase in temperature, early termination of diapauses and shortening of development time, more number of generations of gram pod borer are predicted (Sharma, 2005, 2010). This warrants engineering of broad spectrum resistance in chickpea for enhanced protection. We engineered a chimeric *Bt* gene (*cry1Aabc*) for improving the level of insect resistance. The chimeric protein differs from Cry1Ac protein (truncated) with respect to five amino acids in domains I and II. The domain shuffled and reconstructed *Bt* gene may delay resistance development in insects more effectively than *cry1Ac*. Among the lepidopteran specific ICPs of Bt, *cry1Ac* class is the most effective toxin against *H. armigera* (Chakrabarti et al., 1998). Attempts to improve the level of toxicity of *cry1Ac* by domain shuffling have led to the finding that a chimera of *cry1Aa*, *cry1Ab*, and *cry1Ac* was more effective than *cry1Ac* (Kumar, unpublished). Further, codon optimization for plant expression was reported to increase translational efficiency and subsequent protein synthesis (Perlak et al., 1991).

Genetic engineering employing ICPs of Bt (*Bacillus thuringiensis*) provide resistance against Lepidopteran insects in crop plants (ISAAA, 2016). Reports of chickpea transformation for insect resistance are abundant in last two decades (Kar et al., 1997; Sarmah et al., 2004; Sanyal et al., 2005; Ignacimuthu and Prakash, 2006; Chakraborty et al., 2009, 2016; Acharjee et al., 2010; Mehrotra et al., 2011; Ganguly et al., 2014). This is the maiden report of using a chimeric *cry1Aabc* gene for engineering insect resistance in the grain legume, chickpea.

The codon-optimized synthetic *cry1Aabc* gene was sub-cloned in binary vector, pBinAR and mobilized into *Agrobacterium tumefaciens* (EHA105) for genetic transformation of chickpea cultivar, DCP92-3. The *desi* cultivar, DCP92-3, is *Fusarium* wilt resistant, semi-spreading, lodging tolerant genotype widely grown in India, particularly North West plain zone, besides being responsive to plant tissue culture. Development of transgenic chickpea is an arduous task involving standardization of various factors that act tandemly during regeneration. *De novo* organogenesis from responding meristematic region, working out the right combination of hormonal trigger to affect multiple shoots induction and optimal selection pressure are crucial to genetic transformation. *In vitro* regenerated shoots should also be rooted to establish complete plants. The grafting procedure adopted in the study is facile and can be adopted routinely for chickpea establishment. Here, we reported direct shoot organogenesis based regeneration system from co-cultivated explants and selection of putative transformed shoots based on antibiotic kanamycin monosulfate (100 mg/L) (Rizvi, 2009). Healthy green shoots surviving 4–5 cycles of regeneration/selection (10–14 days each) were used as scions and grafted onto *wild-type* (non-transformed) rootstock to establish mature fertile plant, after acclimatization. Genetic transformation frequency of 0.076% was calculated based on advancing positive chickpea lines across generations from the initial number of putative transgenic chickpea lines generated/established. Seeds from all PCR positive putative primary transgenic lines were harvested and sown in Containment facility for generation advancement. Based on extensive molecular analyses and insect bioassays, three independent chickpea events were selected for study (T_2_ generation onward). The events have single locus integration in the genome, high expression (7.96–12.86 ng/mg of TSP) and high insect mortality (up to 100%) based on detached leaf bioassay at T_4_ generation. PCR and Southern blot hybridization confirmed stable single locus integration and transmission of *cry1Aabc* gene to the progenies. Restriction digestion release of the *cry1Aabc* fragment (2.62 kb) indicated intactness of the gene in the genome of the events.

Protein expression studies using different plant tissues collected at different developmental stages of three chickpea events indicated expression variation as reported earlier in cotton (Dong and Li, 2007), chickpea (Acharjee et al., 2010), and pigeonpea (Das et al., 2016). In leaf tissues, protein expression during pre-flowering stage was higher as compared to the post-flowering stage. Expression of Cry1Aabc protein in pod wall and immature seeds were also estimated, since pod borer larvae devour these organs, during reproductive phase. Expression was higher in pod wall as compared to immature seeds. Decreasing Cry1Ac expression levels throughout the growing season were also reported earlier, attributing part of the decline to reduction in the levels of mRNA production (Finnegan et al., 1998). The protein content in specified tissue concomitantly decreased along with the growth of transgenic plants, attributing to the decrease in full length *Bt* toxin gene transcripts. The higher expression at earlier stages led to the gene regulation at the post-transcriptional level and contributed to the consequent gene silencing that was developmentally regulated (Dong and Li, 2007). Lower expression level of the *Bt* insecticidal protein at late developmental stages was also correlated with changes in the methylation pattern of cauliflower mosaic virus 35S promoter (Xia et al., 2005). Lower expression in floral bud on maturity was also attributed to protein degradation (Szwacka et al., 2009).

Efficacy of the transgenic chickpea events were established based on protein expression values (estimated using quantitative ELISA) and insect mortality data across generations. The variable insect mortality levels corroborated mostly with the protein expression levels in the transgenic chickpea progenies of the two events. However, in few transgenic progenies derived from IPCa3 exhibited lower larval mortality, despite higher expression levels. This may be possibly due to altered level of secondary metabolites *viz.* terpenoids, phenolics, etc. as the plant matures (Zummo et al., 1984) or interaction of secondary bioproducts to alter the toxicity positively (Zhang et al., 2002) or negatively (Olsen and Daly, 2000; Zhang and Guo, 2000). Further, higher CO_2_ concentration may also affect efficacy as reported earlier (Chen et al., 2005).

Ideally, transgenic plants with single locus integration should segregate in Mendelian 3:1 ratio among the selfed progenies. Based on segregation analyses of the progenies of three events, it is apparent that the lines were in hemizygous state, with distorted segregation ratio in selfed progenies. This may possibly be due to insertion of the transgene in active coding region of genome, disrupting gene function essential for growth and development. Report of disruption of *OsAux1* gene in transgenic rice lead to abnormalities in the growth and development of transgenic rice lines homozygous for the transgene (Bollinedi et al., 2017). Further, the severity of phenotype appeared to be related to the level of Cry1Aabc protein in transgenic chickpea lines, rather than somaclonal variants. Transgenic chickpea lines expressing higher level of Cry1Aabc protein exhibited developmental and morphological defects such as stunted growth and less seed set. It appears that high level of Cry1Aabc protein is causing growth reduction in chickpea, the nature of which is still unclear. Similar findings were also reported earlier in other crops (Larkin and Scowcroft, 1981; Gahakwa et al., 2000; Acharjee et al., 2010).

Non-Mendelian segregation pattern has been reported earlier in various crops *viz*. transgenic wheat (Srivastava et al., 1996), cotton (Sachs et al., 1998), rice (Datta et al., 1990; Pemg et al., 1995), and soybean (Christou et al., 1989). The distorted segregation pattern may be due to reduced viability of pollen affecting fertilization ability (Christou et al., 1989; Zhang et al., 1996), transgene inactivation (Spencer et al., 1992; Walters et al., 1992), recessive lethal gene action (Scott et al., 1998) or other intra genomic conflicts. Our study on pollen viability and germination of transgenic chickpea lines indicate no discernable difference as compared to non-transgenic control chickpea line (unpublished data). Further research and flanking sequence analyses of the site of integration are required to understand the segregation distortion pattern of chickpea progenies.

Insect bioassays were performed using two techniques *viz*. detached leaf and whole plant bioassay in higher expressing chickpea progenies, using 24 h old neonate from homogeneous brood. Adult pod borers were collected from chickpea field and artificially bred to raise homogenous population for the bioassay, obviating external factors. Larval mortality was documented in the progenies within 7 days of incubation. Further, larvae fed on transgenic plants stopped feeding and most of the tissues remained unaffected, whereas the larvae on untransformed plants fed voraciously, skeletanizing the leaves. The larvae surviving on the transgenic lines were deformed with significantly reduced body weight and hardly advancing beyond molting stage. However, for more effective control of the pod borer, pod wall specific promoter may be utilized (Ganguly et al., 2014).

In the present investigation, we demonstrated that the chimeric Cry1Aabc protein is effective against *H. armigera* larvae in insect bioassays carried across generations. However, the phenotype observed needs further research to understand the exact nature and cause of the reduction in plant growth and productivity in higher expressing chickpea lines and distorted segregation ratio. These events will be further tested to identify a promising event that can be incorporated in breeding programs for the development of insect resistant chickpea.

## Author Contributions

AD, SD, GS, SC, and NS: planned the experiments; AD, SD, GS, AS, JA, and ST: conducted the experiments; AD, GS, AS, ST, PK, and NS: analyzed the data and compiled, reviewed and edited the manuscript.

## Conflict of Interest Statement

The authors declare that the research was conducted in the absence of any commercial or financial relationships that could be construed as a potential conflict of interest.

## Supplementary Material

**FIGURE S1 |** T-DNA of pBinAR containing *cry1Aabc* gene (RB, right border; P*nos*, nopaline synthase promoter; *nptII*, neomycin phospho transferase II; T*nos*, nopaline synthase terminator; T*ocs*, octopine synthase terminator; *cry1Aabc*, domain shuffled *Bt* gene; P35S, Cauliflower Mosaic Virus 35S promoter; LB, left border).

**FIGURE S2 |***Agrobacterium* mediated genetic transformation of chickpea. **(A)** Germinated seedlings of chickpea; **(B)** decapitated shoots and roots; **(C)** axillary meristem explant (AME); **(D)** induction of multiple shoots after co-cultivation; **(E)** elongation of multiple shoots; **(F)** separation of elongated shoots; **(G)** grafting; **(H)** hardening of grafts; **(I)** acclimatized plants; and **(J)** mature fertile plant.
