# Supplementary material for: Expression of a Chimeric Gene Encoding Insecticidal Crystal Protein Cry1Aabc of Bacillus thuringiensis in Chickpea (Cicer arietinum L.) Confers Resistance to Gram Pod Borer (Helicoverpa armigera Hubner.)
Source: Front Plant Sci. 2017 Aug 21;8:1423. doi: 10.3389/fpls.2017.01423 (PMC5566580; doi:10.3389/fpls.2017.01423)
Supplement: Supplementary file 1 [file Table_1.DOC]

**Supplementary Table 1**

| **Sequence Motifs/Elements** | **Cry1Aa**  **(Domain-I)** | **Cry1Ab**  **(Domain-II)** | **Cry1Ac**  **(Domain-III)** | **Cry1Aabc** |
| --- | --- | --- | --- | --- |
| Cryptic Splice Sites | 1 | 1 | 2 | - |
| Splicing Enhancer elements | 9 | 6 | 11 | 3 |
| PolyAdynelation sites | | | | |
| ATTAAA | - | - | 1 | - |
| AGTAAA | 1 | 1 | - | - |
| GATAAA | - | 1 | - | - |
| AATATA | - | 1 | 2 | - |
| AATACA | - | - | 1 |  |
| UTR Motifs | 1 | 4 | 4 | 3 |
| AU rich Elements | 10 | 16 | 11 | 2 |

**Comparison of different negative regulatory elements present in the native and codon optimized sequences of Insecticidal Crystal Protein (ICP) gene**

**Supplementary Table 2**

**Whole Plant Bioassay (t- test)**

# Analysis of Variance table

| **Source of Variation** | **d.f.** | **Mean Squares** | **F-Calculated** | **Significance** |
| --- | --- | --- | --- | --- |
| Treatment | 1 | 18,984.375 | 36.954 | 0.00000 |
| Error | 22 | 513.731 |  |  |
| Total | 23 |  |  |  |

# Tables of Mean, Standard Errors and CD

| **Treatment** | **Mean** | **S.E.** |
| --- | --- | --- |
| IPCa2 | 60.417 | 8.950 |
| Control | 4.167 | 2.350 |
| C.D. | 19.314 |  |
| SE(m) | 6.543 |  |
| SE(d) | 9.253 |  |
| C.V. | 70.190 |  |
