# Supplementary material for: Expression of a Chimeric Gene Encoding Insecticidal Crystal Protein Cry1Aabc of Bacillus thuringiensis in Chickpea (Cicer arietinum L.) Confers Resistance to Gram Pod Borer (Helicoverpa armigera Hubner.)
Source: Front Plant Sci. 2017 Aug 21;8:1423. doi: 10.3389/fpls.2017.01423 (PMC5566580; doi:10.3389/fpls.2017.01423)
Supplement: Supplementary file 2 [file Presentation_1.PPT]

## Slide 1
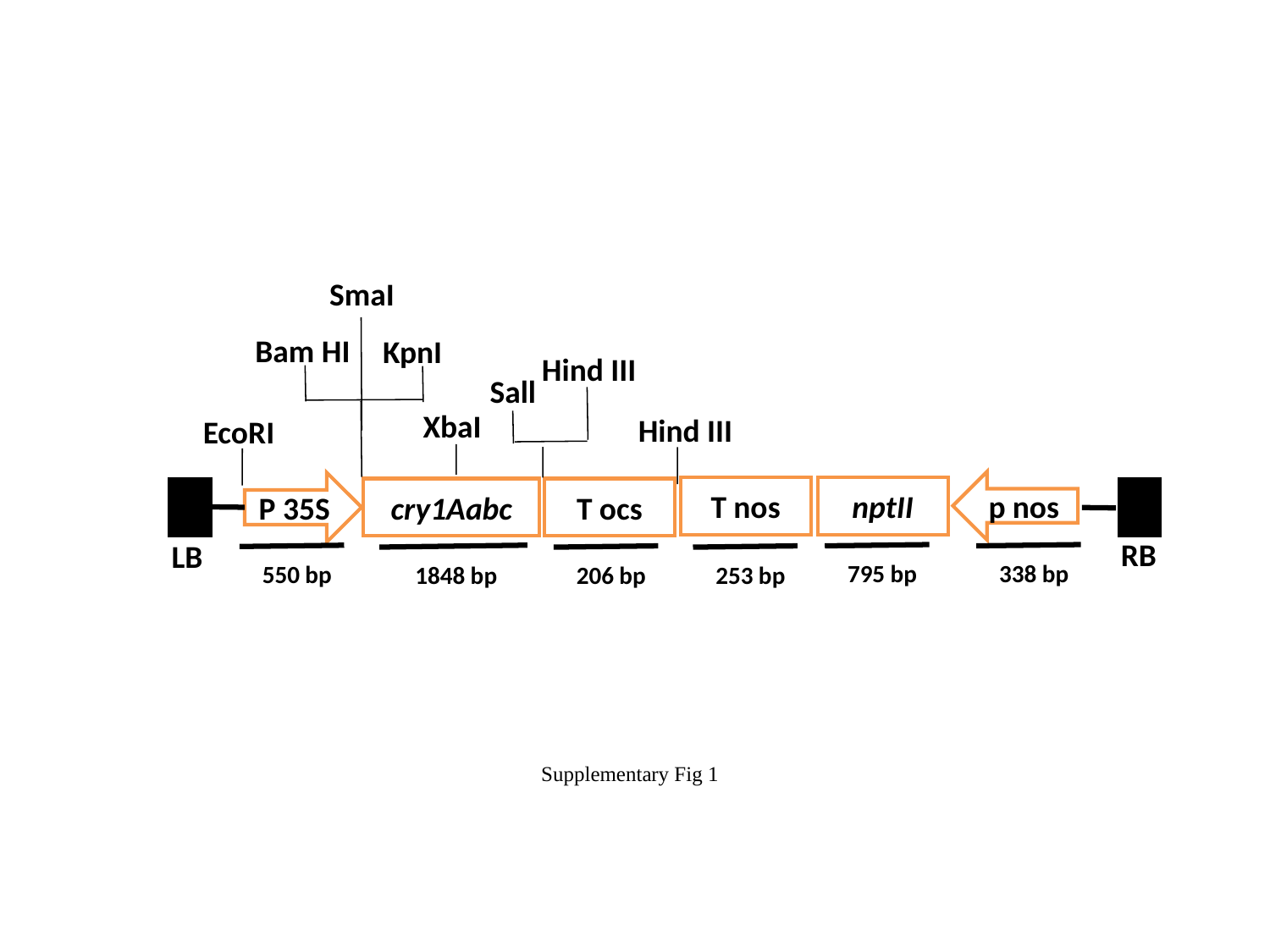

SmaI
Bam HI
KpnI
Hind III
Sall
XbaI
Hind III
EcoRI
p nos
P 35S
T nos
nptII
cry1Aabc
T ocs
RB
LB
795 bp
338 bp
550 bp
1848 bp
206 bp
253 bp
Supplementary Fig 1

## Slide 2
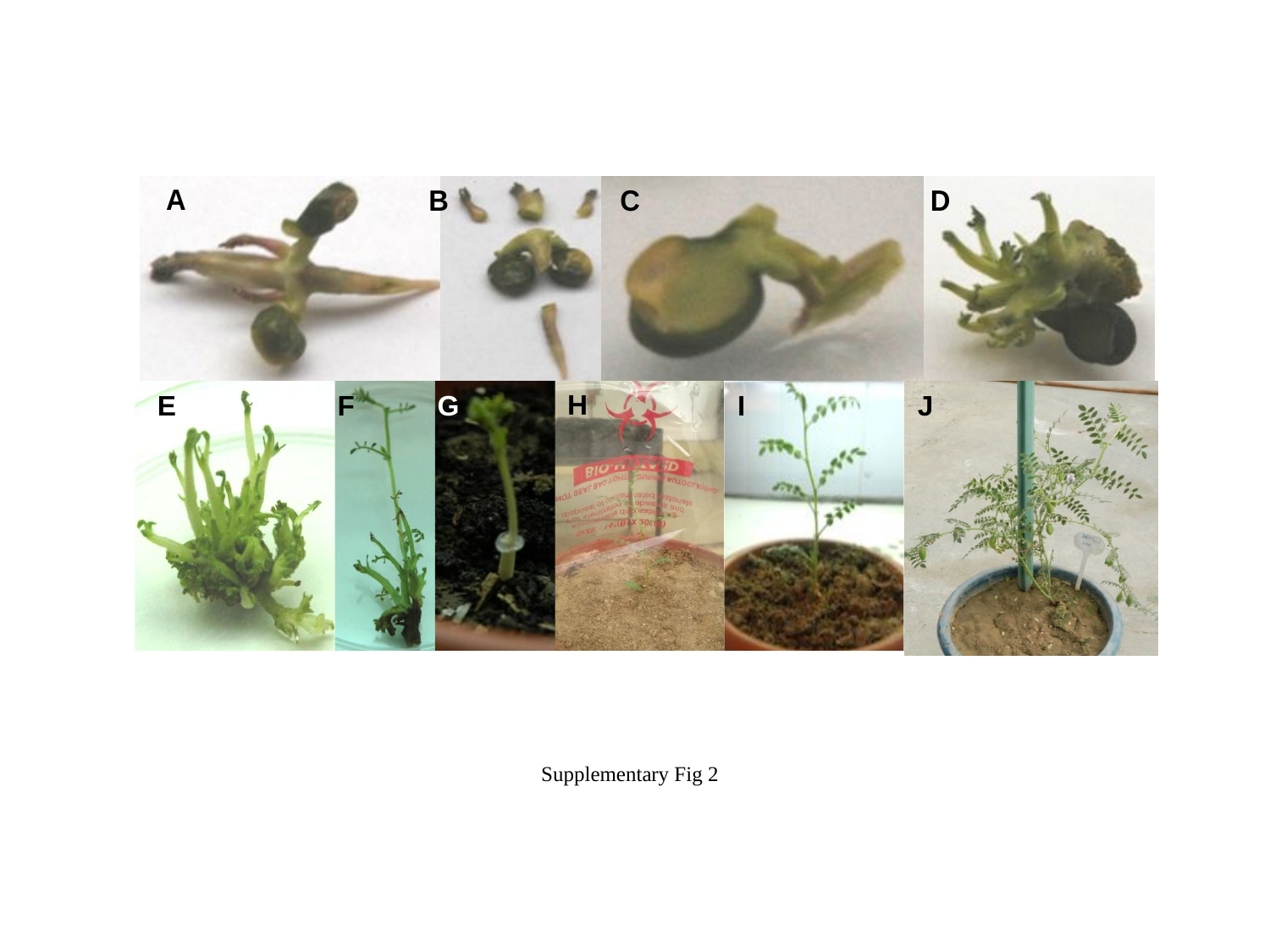

H
E
F
G
I
J
Supplementary Fig 2
